# Effect of a Whole-Body HIIT Session on Psychophysiological Responses, Muscle Damage, and Oxidative Stress in Healthy Young Men: A Pilot Study

**DOI:** 10.3390/sports14100418

**Published:** 2026-09-28

**Authors:** Paulo Vinicios C. Zovico, Pedro Henrique C. Lirio, Lívia Carla de M. Rodrigues, Andre S. Leopoldo, Valentina Bullo, Stefano Gobbo, Marco Bergamin, Roberta L. Rica, Luciene Cristina G. Campos, Valério G. Barauna, Danilo S. Bocalini

**Affiliations:** 1 Experimental Physiology and Biochemistry Laboratory, Physical Education and Sport Center, Federal University of Espírito Santo, Vitoria 29075-910, Espírito Santo, Brazilbocaliniht@hotmail.com (D.S.B.); 2 Laboratory Neurotoxicology and Psychopharmacology, Department of Physiological Sciences, Federal University of Espírito Santo, Vitoria 29043-900, Espírito Santo, Brazil; 3 Department of Medicine, University of Padova, 35128 Padova, Italy; 4 Gymhub S.r.l. Spin-off of the University of Padova, 35129 Padova, Italy; 5 Department of Physical Education, Estacio de Sá University, Vitoria 29092-095, Espírito Santo, Brazil; 6 Laboratory of Applied Pathology and Genetics State, Department of Biological Science State, University of Santa Cruz, Ilheus 45662-900, Bahia, Brazil; 7 Molecular Physiology Laboratory of Exercise Science, Federal University of Espírito Santo, Vitoria 29075-910, Espírito Santo, Brazil

**Keywords:** biomarkers, physical fitness, body weight, recovery, exercise-induced stress

## Abstract

**Introduction:** Optimizing exercise prescription requires a comprehensive understanding of exercise-induced muscle damage (EIMD) kinetics. This study investigated the acute and 72 h recovery responses of psychophysiological markers, muscle damage, and oxidative stress following a single calisthenic high-intensity interval training (HIIT-C) session in healthy young men. **Methods:** Ten physically active healthy young men completed a HIIT-C protocol consisting of a 5 min warm-up followed by four blocks of five 30 s “all-out” bodyweight exercises (jumping jacks, burpees, mountain climbers, and jump squats) interspersed with 30 s passive recovery intervals. Perceptual, cardiovascular, functional, biochemical, oxidative stress, and circulating cell-free DNA (cfDNA) markers were assessed pre-exercise, immediately post-exercise, and at 24, 48, and 72 h post-session. Repeated measures were analyzed using a one-way ANOVA (*p* < 0.05). **Results:** The HIIT-C session elicited significant acute increases in heart rate, blood lactate, and perceived exertion (*p* < 0.001), accompanied by an immediate reduction in perceived recovery. Mood state analysis revealed immediate post-exercise increases in fatigue and decreases in vigor (persisting for 72 h), without alterations in depression and anger. Muscle soreness increased and general well-being decreased up to 48 h post-exercise. Nuclear cfDNA (n-cfDNA) was elevated immediately post-session and returned to baseline within 24 h, whereas mitochondrial cfDNA (mt-cfDNA) remained unchanged. Significant elevations in muscle thickness (biceps brachii, triceps brachii, rectus femoris), serum enzymes (CK, LDH, AST, ALT) and oxidative stress markers (TBARS, AOPP) were observed alongside reductions in the countermovement jump performance and pressure pain threshold. Most markers remained altered at 24 and 48 h; by 72 h post-exercise, all variables returned to baseline values except for the biceps brachii muscle thickness. **Conclusions:** A single HIIT-C session induces significant psychophysiological perturbations and systemic EIMD requiring a 48 to 72 h recovery period. These findings delineate the post-exercise recovery kinetics of bodyweight HIIT, providing empirical data to assist practitioners in optimizing training periodization and mitigating overtraining risks in young adult men.

## 1. Introduction

Perceived lack of time is a primary barrier to physical activity adherence across diverse populations [1,2,3]. High-intensity interval training (HIIT) addresses this limitation by efficiently improving physical fitness compared to traditional continuous training [2,4,5]. Despite some conflicting evidence [6,7,8,9], HIIT generally promotes higher perceived enjoyment [10,11,12,13].

Among different protocols [4,14,15,16], bodyweight HIIT (HIIT-C) has emerged as a major global trend [17] across children, adolescents [18,19], adults [20,21,22,23], and older adults [24] due to its low cost, accessibility, and minimal space requirements. Acutely, HIIT-C elicits robust cardiorespiratory responses (VO^2^ peak and heart rate) similar to sprint protocols [25], alongside increased lactate [20,26,27], perceived exertion [20,26], sweating [26], and caloric expenditure [28]. Chronically, HIIT-C enhances cardiorespiratory fitness [2,29], body composition [20,29], strength performance [2,30,31], muscle thickness [31], and affective responses [32].

However, while fitness outcomes are well documented [2,33], the effects of HIIT-C on exercise-induced muscle damage (EIMD) and oxidative stress remain poorly characterized. A high repetition volume [22,27,34] and metabolic acidosis [23] during HIIT-C are known triggers for EIMD [35,36]. Traditional HIIT studies demonstrate elevated biomarkers such as creatine kinase (CK) [37,38,39,40], lactate dehydrogenase (LDH) [38], myoglobin (Mb) [38], AST [39], and ALT [39] up to 48 h post-exercise [37,38,39,40]. Furthermore, high-intensity exercise stimulates reactive species production [41,42] and antioxidant enzyme activity as a protective mechanism [43], elevating TBARS, protein oxidation, and antioxidant levels [44,45,46]. Concurrently, EIMD [47,48] and oxidative stress [49] trigger the release of circulating cell-free DNA (cfDNA)—an emerging early marker of muscle injury and overtraining [48,50,51].

Understanding EIMD kinetics and post-exercise recovery is critical for designing safer training programs, monitoring athlete readiness [52,53,54], and preventing overtraining or maladaptation [54,55,56]. Given the multifactorial nature of recovery, a continuous evaluation of physiological and psychological markers is needed [54]. Therefore, this study aimed to investigate the acute effects of a single HIIT-C session on psychophysiological markers, muscle damage, and oxidative stress, tracking their recovery kinetics over a 72 h period in healthy young men.

## 2. Material and Methods

### 2.1. Participants

After approval by the Research Ethics Committee for Human Use at Universidade Federal do Espírito Santo, no. 4.212.489, CAAE: 30325420.8.0000.5542, healthy and physically independent adult men were recruited in gyms, using posters, and social media platforms such as Instagram, Facebook, and Twitter. 

The following participants were recruited for inclusion in this study: physically active and independent healthy men aged 20 to 40 years and with a BMI < 30 kg/m^2^. Individuals who had joint and/or skeletal muscle disorders in any limb that prevented the performance of the exercises in the protocol, smokers, those with cardiovascular and metabolic alterations according to ergospirometry, those who used substances such as medications and/or supplements, those who marked “YES” in the Physical Activity Readiness Questionnaire, those who used buffer substances or creatine as supplements or any ergogenic resource in the study period, and those failed to agree with the provided informed consent form were excluded from this study. Individuals who did not complete all assessments were also excluded from the analysis. 

Finally, the remaining 10 subjects and as part of the experimental control protocol, participants were explicitly instructed to abstain from alcohol and caffeine consumption, as well as from engaging in vigorous physical exercise, during the 48 h prior to baseline testing. Moreover, participants were instructed to maintain their usual meals for 24 h before the interventions and to arrive at the laboratory well-fed. 

### 2.2. Anthropometric Evaluation

To characterize the sample, anthropometric evaluations, a maximum oxygen consumption assessment via a cardiopulmonary test, and a physical activity level test were performed. The characteristics of the participants are described in Table 1.

The anthropometric parameters were assessed according to previously described procedures [57]. Height was measured using a Cardiomed stadiometer (WCS model; range: 115–220 cm), with participants standing barefoot, feet together, and the head positioned in the Frankfurt horizontal plane. Body mass was measured using a digital anthropometric scale (Marte Scientific, L200, São Paulo, SP, Brazil), and body mass index (BMI) was subsequently calculated. Body fat percentage (BF%) was estimated from seven skinfold thickness measurements obtained at the subscapular, triceps, mid-axillary, pectoral, abdominal, suprailiac, and mid-thigh sites using a Cescorf skinfold caliper (Mitutoyo Cescorf, Porto Alegre, Brazil).

### 2.3. Evaluation of Maximum Oxygen Uptake

To evaluate maximum oxygen uptake, a maximal effort test was performed on a treadmill (Inbra Sports Super, ATL, Porto Alegre, RS, Brazil) with a 1% slope in the ramp protocol that lasted from 8 to 12 min to determine the maximum speed (vVO_2max_), heart rate (HR_max_), and oxygen uptake (VO_2max_) according Neves et al. [57]. The test began with the subjects running at 5.0 km/h with gradual 1.0 km/h speed increments every minute up to voluntary exhaustion. The procedure was supervised by a cardiologist (room temperature: 23–25 °C). The active recovery phase lasted five minutes. Subjects’ perceived exertion was quantified at each stage of the ergospirometry test based on the Borg gradual 10-point linear scale (0 to 10) fixed near the treadmill. Electrocardiographic recordings were made before the test as patients stood on the treadmill and throughout the exercise (Micromed USB electrocardiograph, Brasília, Brazil). At least four criteria were considered to define the cardiopulmonary test as the maximum test: (a) voluntary exhaustion; (b) ≥90% of the estimated age-adjusted HR_max_; (c) z-respiratory exchange ratio equal to or above 1.05; and (d) VO_2max_ plateau with increased exercise intensity. During the test, a metabolic gas analyzer (Cortex Metalyzer 3B, Leipzig, Germany) was used with breath-by-breath measurements. Before each test, a gas mixture was used to calibrate the ambient gas (11.97% O_2_ and 4.95% CO_2_). 

### 2.4. Assessment of Physical Activity Level

The physical activity level was determined using the short version of the International Physical Activity Questionnaire, as described by Machado et al. [21,28] and Filho et al. [23]. In this study, the total time of weekly physical activity was used to characterize individuals as active (those who performed more than 150 min per week of physical activity) and inactive (those who performed less than 150 min per week of physical activity).

### 2.5. Experimental Design

The sample size was estimated at 18 participants, based on an analysis using G Power (v.3.1.9.4) for a power of (1-beta) = 0.95 and alpha = 0.05, with an effect size of 0.45. However, to mitigate sample loss, 20 individuals were chosen. After applying the inclusion and exclusion criteria, 24 volunteers were recruited for this study. However, three withdrew after the initial evaluation of this research for personal reasons, three others reported complications during blood collection, and eight failed to follow the procedures due to problems encountered during the project, including equipment availability, laboratory issues, and a lack of qualified personnel to collect blood samples, among others. 

The study was conducted across five visits to the Laboratory of Physiology and Experimental Biochemistry at the Federal University of Espírito Santo (UFES), structured as follows above and showed in Figure 1:

- Visit 1 (Screening and Familiarization): Completion of informed consent and eligibility questionnaires, anthropometric assessments, a clinical maximal effort test, familiarization with the HIIT-C exercise protocol, and instruction on measurement scales.
- Visit 2 (Intervention and Baseline Testing): Execution of the bodyweight HIIT exercise protocol alongside comprehensive physiological and psychometric evaluations (heart rate, blood lactate, repetition count, perceived exertion, pain, recovery, Brunel mood scale, blood sampling, muscle thickness, countermovement jump, among others).
- Visits 3, 4, and 5 (Follow-up Assessments): Re-evaluation using the same battery of physical, physiological, and psychological assessments from the second visit to track recovery and physiological responses over time.

Compliance with these guidelines was verified through self-reports and verbal declarations from the volunteers upon arrival at the laboratory. Additionally, all study participants were explicitly instructed to maintain their standard, habitual diet in the days leading up to the test, with no specific pre-test meal recommendations or standardized diets provided by the researchers. Consequently, decisions regarding meal timing, total caloric intake, and carbohydrate consumption were left to the individual discretion of each participant, provided that they adhered to their usual eating patterns. The only mandatory dietary restrictions enforced by the experimental protocol were the total abstinence from alcohol and caffeine for the 48 h prior to testing.

### 2.6. Exercise Protocol

The volunteers were previously familiarized by performing a set for each exercise that they had learned a week prior. The HIIT-C session consisted of a standard five-minute warm-up (one minute of stationary running, 30 s of jumping jacks, burpees, mountain climbing, jump squats, and another minute of walking). The HIIT-C protocol consisted of 20 sets organized into four exercise blocks, with each block comprising five 30 s exercise bouts interspersed with 30 s of passive recovery. The exercises were performed in the following order, as previously described in the literature: jumping jacks, burpees, mountain climbers, and jump squats [21,22,23,27,58]. The session was conducted with an “all-out” intensity and monitored using the adapted Borg scale (0–10) immediately after each set and the recovery scale (0–10) in the final 10 s of recovery from the sets.

The following variables were analyzed before, after, and 24, 48, and 72 h after the session: number of movements, heart rate, lactate, perceived effort, perceived recovery, visual analog scale for pain perception, pressure pain threshold, muscle circumference, muscle thickness, countermovement jumping, blood collection, pleasure perception scale, Brunel mood scale, recovery scale, and the well-being questionnaire (Figure 1). 

### 2.7. Evaluated Parameters

To facilitate an understanding of this study’s proposal, the evaluated parameters will be divided into two categories: acute and subacute parameters (psychophysiological parameters assessed only during or immediately after the HIIT-C session) and those indicating muscle damage (psychophysiological and biochemical parameters measured immediately after the session and at 24, 48, and 72 h afterward).

#### 2.7.1. Heart Rate

Heart rate (HR) was continuously monitored throughout the session and recorded after each exercise bout and at the end of the session using a Polar heart rate monitor (S810 model), as previously described [27]. To minimize potential measurement errors, participants were instructed to check the positioning and attachment of the chest strap 10 min before the beginning of the protocol. Relative heart rate (%HRmax) during the exercise protocol was calculated based on the maximal heart rate (HRmax) obtained during the cardiopulmonary exercise test.

#### 2.7.2. Lactate Concentration

A sterile lancet was used to take capillary blood samples from the subjects’ properly sterilized fingers to measure blood lactate levels. The first drop of blood was discarded, and the subsequent flowing blood was collected in glass capillary tubes. All samples for lactate analysis were tested using an Accutrend device (Roche, Basel, Switzerland), which had been calibrated according to previous studies [22,23,58].

#### 2.7.3. Number of Movements in the Session

During the exercise session, the total number of movements in each exercise series was counted and recorded as a performance indicator. To quantify movements, a tactile digital movement counter was used, counting the number of movements for each exercise series, as per previous publications from our group [21,22,23,27,58].

#### 2.7.4. Perceived Exertion and Recovery

At the end of each stimulus period, participants’ rate of perceived exertion (RPE) was evaluated and recorded using the Borg 0–10 scale, as adapted by Foster et al. [59]. The scale was shown, and the classification and its respective descriptor for each were explained to volunteers. It ranges from 0 to 10, in which “0” refers to rest and “10” to maximum effort at that time. The following question was asked aloud immediately after each exercise: “What is your perceived effort at this moment?” The scale was shown to participants 10 min after the session, who were asked the following question: “Considering the entire exercise session, what is your perceived effort of the session?”

Immediately before the start of the series, volunteers were asked to rate their subjective perceived recovery (SPR). The scale ranges from 0 to 10, in which “0” indicates “very poorly recovered/extremely tired” and “10” indicates “very well recovered/highly energetic.” This classification was applied during recovery between sets and 10 min after the end of the session (as in previous studies) [27]. A score of 0, 1, or 2 is associated with an expected decrease in performance, whereas a score of 8.9 or 10 suggests an expected improvement in performance. The range of values from 3 to 7 indicates no changes in expected performance [55].

#### 2.7.5. Perceived Pleasure

The pleasure scale (feeling scale) was used to assess perceived pleasure, and an 11-point bipolar scale ranging from +5 to −5 was commonly used to measure affective responses (pleasure versus discontent) during exercise. This scale has the following verbal anchors: −5 = very poor, −3 = bad, −1 = quite bad, 0 = neutral, +1 quite good, +3 = good, and +5 = very good. Previous studies have recommended this scale to measure affective responses to exercise [60]. Subjects received standard guidance on using the scale in their preliminary instructions, before the use of the scale, immediately after it, and 24, 48, and 72 h later. The scale was shown to volunteers, who chose a “descriptor/number” that represented their feeling at that moment.

#### 2.7.6. Brunel Mood Scale

Mood was assessed using the Brunel Mood Scale before the session, immediately after it, and at 24, 48, and 72 h later. The following question was asked: “How do you feel now?” The instrument assesses six subjective mood states: tension (T, items: panicky, anxious, worried, and mixed-up), depression (D, items: depressed, sad, unhappy, and discouraged), anger (R, items: annoyed, angry, bad tempered, and nervous), vigor (V, items: lively, active, energetic, and alert), fatigue (F, items: worn out, exhausted, sleepy, and tired), and mental confusion (C, items: confused, uncertain, muddled, and downhearted). Factors T, D, R, F, and C are considered negative, and vigor is deemed positive. Total mood disturbance was calculated by subtracting the sum of the negative factors from the sum of the positive aspects (DTH = (T + D + R + F + C) − V), thereby configuring a global measure of mood disturbance. To it, 100 was added to avoid negative results. The mood profile with a high vigor value and low values for the other variables is referred to as the “iceberg profile,” representing positive mental health. Each participant was instructed to answer according to how they were feeling “now, at this very moment” when responding to the scale [23,61].

#### 2.7.7. Well-Being Questionnaire

The well-being questionnaire evaluates general well-being across five domains: fatigue, sleep quality, general muscle pain, stress, and mood levels. Volunteers were to rate their current level on a scale of 1 to 5. Overall well-being is then determined based on the sum of the five scores [62,63].

### 2.8. Parameters Indicating Muscle Damage

#### 2.8.1. Visual Analog Scale

To assess pain perception, the visual analog scale (VAS) was used, as per previous studies [64], where “0” corresponded to “no pain” and 10 corresponded to “extreme or maximum pain.” Participants received standard instructions in advance and on the day before the exercise session.

#### 2.8.2. Pressure Pain Threshold

The pressure pain threshold (PPT) of the biceps brachii, triceps brachii, and rectus femoris was determined using a pressure algometer (Wagner Instruments, Greenwich, CT, USA, FPX 50/220). PPT was shown in Lbs based on the pressure required to induce discomfort in the muscle. The algometer was positioned perpendicular to the middle portion of the muscle, and force was gradually applied until the participant reported the first noticeable sensation of pain. The value on the algometer constituted the PPT [65].

#### 2.8.3. Muscle Circumference

Limb circumference was evaluated at the midpoint of the biceps brachii (between the acromial process and the lateral epicondyle of the humerus, measured in cm), with volunteers’ arms relaxed along their body and their arms contracted. Leg circumference was assessed at the midpoint of the quadriceps at a distance from the perimeter of the thigh perpendicular to the long axis of the femur at the level of the lateral trochanteric, median tibial, which was marked and assessed. The assessment was also performed with the leg in a contracted position. The subjects stood with their feet about 10 cm apart, with their body weight evenly distributed across their feet. This measurement was performed three times, and the mean of these measurements was used as the limb circumference [66,67].

#### 2.8.4. Muscle Thickness

Using portable equipment (BodyMetrix, BX2000, IntelaMetrix, Inc., Livermore, CA, USA), ultrasonography was used to obtain muscle thickness (MT) measurements of volunteers’ biceps brachii and triceps brachii at 60% of the measurement between the acromion of the scapula and the epicondyle of the humerus [31]. The rectus femoris was measured at a 50% distance between the greater trochanter and the lateral condyle of the femur [31]. The measurements were taken from the right side of the body in a single-dimensional mode, as per previous studies [31,68,69], and were used as markers of muscle damage in accordance with Evangelista et al. [58]. Muscle thickness was assessed before the session, immediately after it, and 24, 48, and 72 h later.

A water-soluble transmission gel (Aquasonic 100 Ultrasound Transmission Gel, Parker Laboratories Inc., Fairfield, NJ, USA) was applied to each measurement site, and a 2.5 MHz ultrasound wave was applied perpendicular to the skin relative to the measurement site. When the image quality was deemed satisfactory, the image was saved to obtain the dimensions of muscle thickness, as reported in previous publications [31,68,69].

Sites were measured with a vinyl measuring tape and then marked with henna to ensure accuracy between measurements throughout the protocol. During upper extremity measurements, participants remained standing with their arms relaxed. In measuring their lower limbs, individuals remained standing, with their body weight distributed across their lower segments. To ensure accurate measurements, three measurements were taken from each segment. A fourth measurement was taken in cases where the difference was greater than 10%. The mean of the three measurements for each muscle group was used in this study.

To standardize and analyze the reliability of the image acquisition and analysis process, 10 trained individuals underwent two evaluations in accordance with the procedures outlined above to validate the methods used in this study. The intrarater review found the following measurement errors: biceps brachii (0.63 ± 2.12 mm; 95% CI: −0.16 to 1.42), triceps brachii (0.35 ± 1.16 mm; 95% CI: −0.07 to 0.78), and rectus femoris (0.19 ± 0.91 mm; 95% CI: −0.14 to 0.53).

#### 2.8.5. Countermovement Jumping

Countermovement jumps serve as a parameter of physical performance [48]. Countermovement jumps were used to assess EIMD indirectly. For this, all participants stood and were asked to jump as high as possible with an eccentric action, as described in previous publications [48,70]. All jumps were performed keeping the hands on the iliac crest to avoid the influence of the upper limbs on the jumping performance. In total, five countermovement jumps were performed in 12 s intervals. All jumps were performed on a mat and measured based on the Jump Test System (Belo Horizonte, MG-Brazil). The best attempt and the average of the five attempts were repeated for further analysis.

#### 2.8.6. Blood Collection

For muscle damage marker, oxidative stress, and cfDNA analyses, blood samples were collected under the hygienic conditions required by public agencies, obtained by a trained and experienced professional affiliated with the Federal University of Espírito Santo. Blood samples were collected at different times: before, after, and 24, 48, and 72 h after the exercise protocol. Collections were carried out at the same time of day in the same environment, with controlled temperature and humidity to avoid any circadian and external interference [71]. About 10 mL of venous blood was collected from the forearm of each participant via a vacuum tube without any anticoagulants. The sample was centrifuged at 1000 *g* for 15 min at 4 °C to obtain the serum. The serum was immediately separated into aliquots and placed in Eppendorf tubes and then stored in a −80 °C freezer for future analysis.

#### 2.8.7. Biochemical Markers of Blood Muscle Damage

To evaluate conventional blood markers such as creatine kinase (CK), cardiac creatine kinase (CK-MB), lactate dehydrogenase (LDH), aspartate aminotransferase (AST), and alanine aminotransferase (ALT), commercial Bioclin kits were used according to the manufacturer’s guidelines and following previous publications by our group [48,66].

#### 2.8.8. Evaluation of Oxidative Stress

For oxidative stress analysis, lipid peroxidation levels were evaluated using the thiobarbituric acid reactive substance (TBARS) assay, and for protein oxidation, the advanced protein oxidation products (AOPP) assay was employed following previous publications from our group. All samples were taken in triplicate. Results are shown in μmol/μL [72,73,74]. 

### 2.9. Cell-Free DNA Analytical Assay 

#### qPCR Analysis

qPCR was performed using a QuantStudio 3 (Thermo Fisher Scientific, Massachusetts, USA). To quantify cell-free nuclear DNA (n-cfDNA), we used primers targeting L1PA2_90_ and L1PA2_222_: forward (5′-TGCCGCAATAAACATACGTG-3′) and reverse (5′-GACCCAGCCATCCCATTAC-3′); and L1PA2_222_: forward (5′-TGCCGCAATAAACATACGTG-3′) and reverse (5′-AACAACAGGTGCTGGAGAGG-3′) [75]. For the quantification of cell-free mitochondrial DNA (mt-cfDNA), primers targeting the mitochondrial genome region Mt:15195–15279 were used: forward (5′-CTATCCGCCATCCCATACATTG-3′) and reverse (5′-ATCGTGTGAGGGTGG-GACTG-3′) [76], yielding an amplicon of 85 bp. The protocol followed previous publications from our group [77,78,79].

### 2.10. Statistical Analysis

Data distribution and normality were evaluated using the Shapiro–Wilk test. Descriptive statistics are presented as the mean ± standard deviation (SD), mean differences, and 95% of confidence intervals (95% CI). A one-way repeated-measures (ANOVA) was conducted to evaluate the effect of time across time points (pre-exercise, post-exercise, and 24, 48, and 72 h post-exercise). When a significant main effect of time was detected, Bonferroni-adjusted post-hoc pairwise comparisons were performed. The Hedges’ g was used to assess pairwise effect sizes and qualitative thresholds: 0.2–0.5 as small, 0.5–0.8 as moderate, and >0.8 as large. Statistical analyses were performed using GraphPad Prism (version 8.0.2; GraphPad Software, San Diego, CA, USA). Statistical significance was set at *p* < 0.05.

## 3. Results

### 3.1. Acute and Subacute Parameters

The total number of movements in the session was 591.10 ± 98.21 (CV: 16.48%). Table 2 shows significantly increased HR (58.3%), lactate concentrations (92.5%), and perceived exertion (90.6%) and decreased perceived recovery (94.2%) immediately after the HIIT-C session.

Figure 2 shows the HR values of the session (Panel A) and the exercise blocks (Panel B) during the HIIT-C session. HR values during the series increased significantly (*p* < 0.05) from the 3rd to the 11th set and from the 16th to the 20th set compared to the first set. As in Figure 2B, the average HR during burpee sets (173.7 ± 9.4 bpm) showed higher values than jumping jack (164.3 ± 12.9 bpm, *p* < 0.0001), mountain climbing (164.6 ± 8.5 bpm, *p* < 0.0001), and jump squat ones (170.3 ± 9.9 bpm), resembling those for jump squat sets (170.3 ± 9.8 bpm, *p* < 0.0001). Participants’ HR during jump squats exceeded that of jumping jacks (*p* = 0.0015) and mountain climbing (*p* < 0.0001), showing similar values (*p* = 0.99) between jumping jacks and mountain climbing.

RPE increased significantly (*p* < 0.0001) from the 10th set up to the end of the HIIT-C session (Figure 2B). Considering the mean RPE during the exercise blocks (Figure 2C), that with jumping jacks (3.3 ± 1.7) showed lower values than with burpees (6.9 ± 1.8, *p* = 0.0002), mountain climbing (9 ± 1, *p* < 0.0001), and jump squats (9.6 ± 0.5, *p* < 0.0001). Burpees showed lower RPE values (*p* < 0.05) than those for mountain climbing and jump squat blocks (which failed to differ from each other). 

Regarding SPR (Figure 2E), values showed an expected inverse relation to RPE. This study found a significant reduction (*p* < 0.0001) in SPR after the sixth set, remaining so after the end of the HIIT-C session. The SPR of the exercise block containing burpees (2.8 ± 2.1), mountain climbing (1.4 ± 0.9), and jump squats (0.6 ± 0.7) remained lower (*p* < 0.0001) than the block containing jumping jacks (6.2 ± 2.4) (Figure 2F). This study found no differences (*p* > 0.05) between the burpees and mountain climbing blocks. Moreover, only burpees differed (*p* < 0.0001) from the jump squat block. 

### 3.2. Perceived Mood, Well-Being, and Recovery

Table 3 shows parameters related to perception, mood, and well-being. This study found no significant changes in depression and anger, observing significantly reduced tension at 72 h and vigor at 48 h and an increased perceived fatigue after the HIIT-C session. Perceived fatigue, mental confusion, and mood disturbance differed significantly (*p* < 0.05) at 24 and 48 h. Moreover, fatigue and mood disturbance remained altered 72 h after the session compared to after. No significant changes occurred in quality of sleep, stress levels, and mood. However, fatigue, muscle soreness, and general well-being showed a significant difference after the HIIT-C session for fatigue. Fatigue, muscle soreness, and general well-being significantly differed from before at the moment only 48 h after the session.

Figure 3A shows subjects’ recovery before, after, and 24, 48, and 72 h after the HIIT-C session. The figure evinces a decreased (*p* = 0.023) perceived recovery immediately (5.2 ± 2.2) after the HIIT-C session in relation to before (7.8 ± 1.6). This study found no significant changes (*p* > 0.05) after 24 (6.5 ± 2.1) or at 48 h (5.5 ± 2.4). However, recovery values after 72 h (7.6 ± 1.6) were higher (*p* = 0.031) than those immediately after the session and similar (*p* > 0.05) to before.

Figure 3B shows the values related to the perceived pleasure before, after, and 24, 48, and 72 h after the HIIT-C session. No differences (*p* = 0.56) occurred in the perceived pleasure after the HIIT-C session (before: 2.4 ± 1.9 = after: 1.9 ± 2.2 = 24 h: 2.7 ± 1.2 = 48 h: 2.2 ± 1.3 72 h: 2.6 ± 2.0).

### 3.3. Reported Pain, Perceived Pain, and Pressure Pain Threshold

The evaluation of the parameters of muscle pain over time after the HIIT-C session and participants’ perceived effects asked volunteers to answer the following question: “Are you feeling pain in any region of the body at this time?”. Before, all volunteers reported no pain, but immediately after, 10% of the subjects indicated quadriceps pain. After 24 h of the HIIT-C session, 40% reported no pain, 40% reported pain in their quadriceps, 10% in their deltoids, and 10% in their latissimus dorsi. After 48 h, 20% reported no pain, whereas 50% had pain in their quadriceps, 50% in their deltoids, and 70% in their latissimus dorsi. After 72 h, 70% reported no pain, whereas 30% had pain in their deltoids, 10% in their quadriceps, and 30% in their latissimus dorsi.

This study used a visual analog scale (VAS) to assess volunteers’ perceived muscle pain (Figure 4A,B). For arm pain, a significant increase (*p* < 0.05) was observed immediately after (1.1 ± 1.2) and 24 h (1.3 ± 1.0) after HIIT-C compared with the baseline. These values remained similar at 48 h (1.0 ± 1.2) and 72 h (0.5 ± 0.5). For leg pain, baseline values (0) were significantly lower (*p* < 0.05) than those observed immediately after (2.0 ± 1.3), 24 h (2.7 ± 1.4), 48 h (2.9 ± 2.1), and 72 h (1.8 ± 1.8) after HIIT-C.

Figure 4 shows volunteers’ pain threshold for their biceps (Figure 4C), triceps (Figure 4D), and rectus femoris (Figure 4E). This study found no significant changes (*p* = 0.0801) in the biceps (before: 22.6 ± 3.4, after: 18.8 ± 3.4, 24 h: 19.5 ± 3.9, 48 h: 20.8 ± 3.9, 72 h: 20.6 ± 3.8; Lbs) and triceps pain threshold (pre: 23.4 ± 6.4, post: 21 ± 5, 1, 24 h: 19.7 ± 4.7, 48 h: 20.2 ± 4.1, 72 h: 22.1 ± 4.7; Lbs, *p* > 0.05). Considering the rectus femoris, a reduction (*p* = 0.0106) in the pain threshold occurred only 24 h (28.5 ± 4.6 Lbs) after the HIIT-C session when compared to before (32.1 ± 4.6 Lbs), failing to differ (*p* > 0.05) from after (28.2 ± 3.9 Lbs) and 48 (30.6 ± 3.8 Lbs) and 72 h (31 ± 4.1 Lbs). 

### 3.4. Muscle Circumference and Thickness 

Figure 5 shows the parameters related to arm and thigh circumferences and biceps, triceps, and rectus femoris thickness. No significant differences (*p* = 0.990) occurred in arm circumference before (32.0 ± 5.2 cm), after (32.4 ± 4.8 cm), and at 24 (32.5 ± 5.1 cm), 48 (32.5 ± 5.0 cm), and 72 (32.2 ± 1.6 cm) h. Similar results were found for the leg circumference (*p* = 0.998) before (55.1 ± 6.2 cm), after (55.5 ± 6.0 cm), and 24 (55.9 ± 5.7 cm), 48 (55.6 ± 5.6 cm), and 72 (55.4 ± 5.7 cm) h after the HIIT-C session.

Increases (*p* < 0.0001) in biceps thickness occurred after persisting for over 72 h (before: 27.6 ± 3.9 < after: 28.5 ± 3.8 = 24 h: 29.3 ± 3.9 = 48 h: 28.8 ± 3.7 = 72 h: 28.8 ± 3.7; mm). Significant changes (*p* = 0.0190) occurred in triceps thickness after, persisting for up to 24 h when compared to before (before: 20.3 ± 3.4 < after: 21 ± 3.5 = 24 h: 21.1 ± 3.5; mm), returning to baseline values after 48 (20.7 ± 3.5 mm) and 72 (20.7 ± 3.1 mm) h. Differences (*p* = 0.0031) occurred in the rectus femoris thickness after persisting for up to 48 h (before: 22.8 ± 5.2 = 24 h: 22.8 ± 5.1 = 48 h: 22.9 ± 5.2 mm) in relation to after (21.88 ± 5.3 mm), failing to differ (*p* > 0.05) from the values 72 (22.5 ± 5.2 mm) h after.

### 3.5. Countermovement Jump

Figure 6 presents the countermovement jump performance. A significant reduction in performance (*p* = 0.0004), corresponding to an −13.15 ± 7.77% decrease, was observed immediately after the HIIT-C session. However, CMJ performance had returned to baseline levels at 24 (−3.38 ± 10.26%), 48 (−2.15 ± 8.92%), and 72 (0.45. ± 6.642%) h post-exercise.

### 3.6. Blood Markers of Muscle Damage

This study evaluated blood damage based on creatine kinase (CK), cardiac creatine kinase (CK-MB), lactate dehydrogenase (LDH), aspartate aminotransferase (AST), and alanine aminotransferase (ALT) values (Table 4). Significant changes (*p* < 0.05) occurred in CK, LDH, AST, and ALT levels after. However, after 24 and 48 h, only CK levels increased (*p* < 0.05) when compared to before. The other parameters showed no changes.

### 3.7. Oxidative Stress and Cell-Free DNA

The oxidative stress TBARS and AOPP are shown in Figure 7. An increase in TBARS was found (*p* = 0.0091) before, which persisted for up to 24 h (after: 5.9 ± 1.5, 24 h: 5.7 ± 1; μM MDA/μL) when compared to before (4.4 ± 0.7), returning to baseline values at 48 and 72 h (48 h: 4.9 ± 1, 72 h: 4.5 ± 1; μM MDA/μL). Similar results were found for AOPP values (after: 31.7 ± 6.2, 24 h: 29.9 ± 8.6; μM chloramine T/μL, respectively) when compared to before (22.1 ± 3.3), returning to baseline values at 48 and 72 h (48 h: 22.5 ± 6.4, 72 h: 22.7 ± 5.8; μM chloramine T/μL).

This study used the primer for mt-cfDNA (MT) and n-cfDNA (L1PA2_90_ for fragments with 90 base pairs and L1PA2_222_ for fragments with 222 base pairs). We hypothesized that cfDNA may represent an early indicator of the physiological stress and/or exercise-induced muscle damage elicited by the HIIT-C session. As expected, L1PA2_90_-targeted n-cfDNA (Figure 7C) increased (*p* < 0.0001) after (134 ± 44 pg/μl) when compared to before (38 ± 18 pg/μl), returning to baseline values after 24 (35 ± 20 pg/μL), 48 (43 ± 37 pg/μl), and 72 (21 ± 16 pg/μl) h and remaining below after. This study obtained similar results for L1PA2 n-cfDNA_222_ (Figure 7D), which increased (*p* = 0.0007) after (22 ± 3 pg/μL) when compared to before (11 ± 5 pg/μL), returning to baseline values after 24 (12 ± 8 pg/μL), 48 (6.8 ± 7.6 pg/μL), and 72 (5.5 ± 5.1 pg/μL) h.

## 4. Discussion

This study evaluated acute psychophysiological responses, markers of muscle damage, and oxidative stress following a HIIT-C session in healthy adults, monitoring recovery over 72 h. HIIT-C elicited an immediate increase in fatigue, whereas a significant reduction in vigor became evident at 48 h post-exercise. Notably, most mood-related variables progressively returned to baseline values within 72 h, indicating a transient perturbation of the affective response following the HIIT-C session. Session pleasure stayed between “quite good” and “good.” Well-being and muscle pain worsened at 24–48 h and normalizing by 72 h.

Speculation has suggested cfDNA as a potentially early predictor of EIMD [48] n-cfDNA increased immediately after the session and returned to baseline levels from 24 h post-exercise onward, while mt-cfDNA showed no changes. Muscle damage was confirmed by soreness and a decreased pressure pain threshold, without an increased muscle circumference and thickness, reduced jump performance, and elevated CK, LDH, AST, and ALT levels. CK peaked at 24–48 h, while oxidative stress markers (TBARS, AOPP) increased immediately, persisting for 24 h and normalizing by 48–72 h.

Thus, HIIT-C induced transient psychological, muscular, and biochemical changes peaking within 24 h and resolving by 48–72 h, suggesting at least 72 h of recovery before a new session. The evidence of this study demonstrates that HIIT-C sessions consistently elevate HR to approximately 90.3% of HRmax, meeting established thresholds for high-intensity exercise [80,81]. This HR-response pattern aligns with observations by Rica et al. [22], Machado et al. [28,82], and Filho et al. [23], confirming the substantial cardiovascular demand inherent to this training modality. Importantly, exercise selection modulates this demand, with compound movements like burpees and jump squats generating significantly greater cardiovascular stress than simpler exercises like jumping jacks, attributable to their higher energy expenditure and metabolic requirements [83]. The increased muscle activation during these exercises may enhance vascular conductance in working musculature, thereby increasing peripheral vascular resistance and consequently elevating HR beyond levels observed in less-demanding tasks, in the order hands, larger active muscle mass, higher metabolic demand, cardiovascular strain, and autonomic activation, without invoking an internally inconsistent vascular mechanism.

The rate of perceived exertion (RPE) also emerges as a critical psychophysiological marker in HIIT-C, with studies consistently reporting peak values of 9.6 ± 0.35 based on category–ratio scales, corresponding to “very difficult/maximum” intensity [22,23,27,28,82]. This aligns with the all-out intensity protocols recommended for HIIT-C, which involve executing maximal repetitions within prescribed work intervals [2,83]. RPE functions as an integrative psychobiological regulator that synthesizes afferent feedback from cardiorespiratory, metabolic, and thermoregulatory systems, enabling individuals to consciously evaluate exercise difficulty through interoceptive awareness [84,85]. The characteristic progressive rise in RPE across HIIT-C sets—particularly pronounced during metabolically demanding exercises like burpees and jump squats compared to jumping jacks—likely reflects accumulated neuromuscular fatigue rather than movement complexity alone [27], though mechanistic studies are warranted to confirm this hypothesis.

Concomitant with the rising RPE, the SPR demonstrates an inverse temporal pattern, declining progressively from the sixth to twentieth sets in standard HIIT-C protocols [22,23,27,28,82]. This SPR attenuation appears to be intimately linked to the recovery interval duration, indicating significantly lower SPR values during protocols featuring 15–30 s inter-set rest periods compared to 60 s intervals. The insufficient recovery time characteristic of shorter intervals sustains elevated percentages of HR and compromises the intramuscular pH restoration [86,87,88], thereby amplifying perceived fatigue. Metabolic data corroborate this through consistently high blood lactate concentrations during HIIT-C, ranging from 12.45 to 15.60 mmol/L [22,23,27]. These levels are commonly associated with voluntary exhaustion and maximal glycolytic flux in both traditional and bodyweight-based HIIT [37,44,89]. This hyperlactatemia may reflect the metabolic acidosis inherent to repeated high-intensity bouts, which accelerates muscle fatigue development through H^+^ accumulation and phosphocreatine depletion [90,91,92]. The phosphocreatine–ATP depletion observed during short recovery intervals (<3 min) potently stimulates glycolytic metabolism, thereby amplifying lactate production beyond the clearance capacity [27]. Furthermore, the session structure influences lactate accumulation, with block-style protocols generating higher concentrations than circuit formats, potentially due to altered exercise sequencing effects on residual fatigue [82]. Interestingly, pause modality (active vs. passive) demonstrates no differential effect on lactate kinetics [93,94,95], though lactate clearance mechanisms remain inadequately studied in HIIT-C contexts. Evidence suggests that intramuscular lactate metabolism requires recovery intervals exceeding three minutes to establish favorable concentration gradients for uptake by previously active muscles [94,96,97], a timeframe rarely incorporated in standard HIIT-C designs.

The affective valence of HIIT-C presents a complex profile distinct from machine-based HIIT. Unlike treadmill protocols that frequently reduce perceived pleasure due to elevated HR, exertion, and metabolic strain [60,98,99], HIIT-C demonstrates neutral effects on immediate post-session pleasure. This divergence may stem from practical advantages like equipment independence, spatial flexibility, and movement variability. The literature reveals contradictory findings regarding the high-intensity exercise affect, with some studies reporting pleasure enhancement [12,100] and others documenting displeasure [60,61,101], suggesting that session design elements critically modulate responses. Indeed, music integration [23] and strategic exercise sequencing [21] can preserve or enhance pleasure, whereas excessive mechanical monotony may provoke negative affect. The dual-mode theory provides a framework for these observations: HIIT-C’s anaerobic dominance likely intensifies interoceptive signaling to prefrontal and subcortical affective centers, potentially generating active displeasure (distress) distinct from the deactivated pleasure (serenity) associated with moderate continuous training. Despite this, practitioners may perceive intermittent protocols as more enjoyable than continuous exercise due to reduced monotony and work distributions [10]. Nevertheless, affect preservation remains crucial for long-term adherence [81,102], warranting careful protocol personalization.

Mood disturbances constitute another significant psychosocial consideration. HIIT-C consistently reduces vigor while increasing fatigue immediately post-session, with vigor decrements persisting for 24 h [23,61,103]. Rosa et al. [103] further documented increased anger and overall mood disturbances, highlighting the psychological strain of all-out protocols. These responses align with the maximal effort paradigm inherent to HIIT-C, which deliberately maximizes metabolic stress and neuromuscular fatigue [2]. Self-selected intensity typically benefits mood regulation [104], but the prescribed maximal effort appears to override this protective effect. Compound movements engaging large muscle masses—particularly those with eccentric components—exacerbate fatigue-related mood alterations [105], suggesting that exercise selection modulates psychological impact.

General well-being assessments reveal no acute post-session changes, though muscle pain and composite well-being scores decrease transiently at 48 h. This contrasts the CrossFit observations where well-being improved at 72 h post-competition [106], possibly reflecting differential training familiarity or the competitive context. Elite athlete data shows stable well-being (19.4 ± 2.3) and minimal pain (3.4 ± 1.0) during periodized training [107], whereas HIIT-C novices report reduced well-being (16.5 ± 2.9) and increased pain (2.3 ± 1.0) at 48 h, suggesting that adaptation moderates discomfort. Such monitoring proves vital for preventing the overtraining syndrome, as inadequate recovery between high-intensity sessions risks performance decrements, injury, and illness [52,108,109].

HIIT-C causes measurable EIMD through multiple biomarker pathways; taken together, our findings suggest that n-cfDNA may serve as a potential early biomarker of the acute response to HIIT-C and, potentially, EIMD [48,110,111]. Nevertheless, further studies are warranted to determine its sensitivity, temporal kinetics, and clinical utility for monitoring EIMD. Exercise intensity, physical impact, thermal stress, and hypoxia contribute to n-cfDNA release [111], with shorter rest intervals (1 min) generating higher concentrations than longer recoveries (5 min) [112]. mt-cfDNA remains unchanged post-HIIT-C, unlike pathological states where it is elevated in cardiovascular disease, cancer, and psychological stress [113,114,115].

Conventional EIMD markers follow predictable kinetics: CK, LDH, AST, and ALT increase immediately post-HIIT-C, with CK peaking significantly at 24–48 h [40,44,89]. CK-MB stability confirms the absence of cardiac involvement [116,117]. The magnitude of enzyme efflux reflects the sarcolemmal disruption severity [66,118], while AST/ALT elevations implicate skeletal muscle rather than hepatic pathology [119,120]. The training status modulates these responses; moderately untrained individuals exhibit greater CK/Mb elevations than well-trained counterparts following identical protocols [89].

Pain perception manifests heterogeneously across modalities. Visual analog scale documents increased arm pain at 24 h and thigh pain at 24–48 h, resolving by 72 h [37,121]. The pressure pain threshold (PPT) decreases specifically in the quadriceps at 24 h [122], reflecting localized mechanical sensitization. Connective tissue damage contributes to delayed-onset soreness and a restricted range of motion [123,124], though comprehensive pain assessments (well-being questionnaire, VAS, PPT, spontaneous reporting) confirm resolution by 72 h.

Ultrasonography reveals prolonged biceps thickening (72 h), transient triceps elevation (24 h), and rectus femoris thickening (48 h) [58,125,126]. These changes reflect fluid shifts and inflammatory infiltration within damaged tissues [66], though equipment limitations in current studies necessitate confirmation with higher-resolution imaging. Functional impairment appears minimal, with countermovement jump performance recovering fully by 48 h despite persistent structural alterations [56].

Oxidative stress is a key mediator of HIIT-C-induced damage; TBARS and AOPP levels were found to be increased immediately post-session and returned to the baseline by 48 h [44,45]. This transient oxidative burst originates from reactive oxygen species (ROS) generation during mechanical and metabolic stress. Eccentric contractions initiate damage through the mechanical disruption of sarcomeres (particularly Z-disc streaming) and excitation–contraction uncoupling [127,128]. Concurrent metabolic stress—from high ATP turnover, oxygen flux, and lactate accumulation—amplifies ROS production via mitochondrial electron leakage and NADPH oxidase activation [129]. The consequent calcium dysregulation activates calpains and phospholipase A2, further propagating membrane damage and inflammation [35,130]. This cascade culminates in the characteristic enzyme efflux (CK, LDH) and edema formation observed 24–48 h post-exercise [36,53]. Despite this damage, the antioxidant system demonstrates adaptive potential, with evidence suggesting that repeated exposure enhances ROS buffering capacity [131,132].

Comprehensive biomarker monitoring reveals that most psychophysiological parameters (mood, oxidative stress, strength) return to the baseline within 72 h post-HIIT-C in novice practitioners. This recovery timeframe appears sufficient given the resolution of muscle pain, normalization of CK levels, and restoration of functional capacity. However, EIMD marker kinetics—particularly the 24–48-h CK peak—necessitate careful training periodization to avoid cumulative damage. Practitioners should consider implementing at least 72 h between introductory HIIT-C sessions, with closer monitoring required for protocols emphasizing eccentric-loaded movements like jump squats.

Additionally, Moscatelli et al. [133] examined acute responses of salivary orexin, alpha-amylase, and cortisol across exercise modalities characterized by different physiological loads, including HIIT, and demonstrated modality- and time-dependent differences in neuroendocrine and autonomic responses. Although the biomarkers and experimental population differ from those used in the present manuscript, the conceptual connection is relevant, due to the consideration that the Moscatelli study and our data may demonstrate that the physiological consequences of acute high-intensity exercise cannot be adequately represented by a single marker and that different biological systems display different temporal kinetics during recovery.

A primary limitation of the present study is the small sample size, with only 10 of the 18 planned participants completing the protocol, which limits statistical precision and generalizability. Additional limitations include variability in physical fitness, the inclusion of healthy men only, a lack of strict dietary control, the restricted organization and variability of the exercise session, and the absence of rigid control of %HR kinetics during exercise. The use of single-dimensional ultrasound may also have limited the assessment of muscle responses. Furthermore, the absence of a non-exercise control condition makes it difficult to distinguish exercise-related temporal changes from normal biological variation, although the magnitude and timing of several responses support an exercise-related effect. Finally, the large number of outcomes may have increased the risk of false-positive findings despite within-variable post hoc corrections. Thus, future research should recruit larger cohort sizes to achieve greater statistical power and enable more comprehensive conclusions. Additionally, subsequent studies should account for participants’ baseline training status and resistance level, as individual physiological adaptions and stress responses to bodyweight HIIT may vary significantly depending on exercise capacity and training background.

From a practical perspective, HIIT-C remains a time-efficient and equipment-independent modality. Its physiological demands may be optimized through strategic exercise selection and sequencing, adequate recovery intervals, progressive exposure to complex movements, and the individualized monitoring of perceived exertion and recovery. However, the present findings should be interpreted in light of important limitations, including the small final sample (10 of 18 planned participants), restriction to healthy men, variability in physical fitness and training status, absence of a non-exercise control condition, limited dietary control, restricted session organization, variability in exercise selection, lack of continuous control of HR kinetics, and limitations associated with single-dimensional ultrasonography. The large number of outcomes also increases the risk of false-positive findings despite within-variable post hoc corrections. Future studies should therefore include larger and more heterogeneous samples, appropriate control conditions, and stratification according to training status and exercise capacity.

In conclusion, HIIT-C constitutes a potent high-intensity stimulus, characterized by substantial cardiovascular and perceptual demands, marked metabolic stress, transient mood alterations, and measurable EIMD and oxidative stress. The observed responses were predominantly transient, with most variables returning toward the baseline within 48–72 h. These findings support the careful individualization of exercise selection, sequencing, and recovery, rather than the adoption of a universal recovery interval, and highlight the need for further research on chronic adaptations and optimal HIIT-C prescription across different populations.

## Figures and Tables

**Figure 1:**
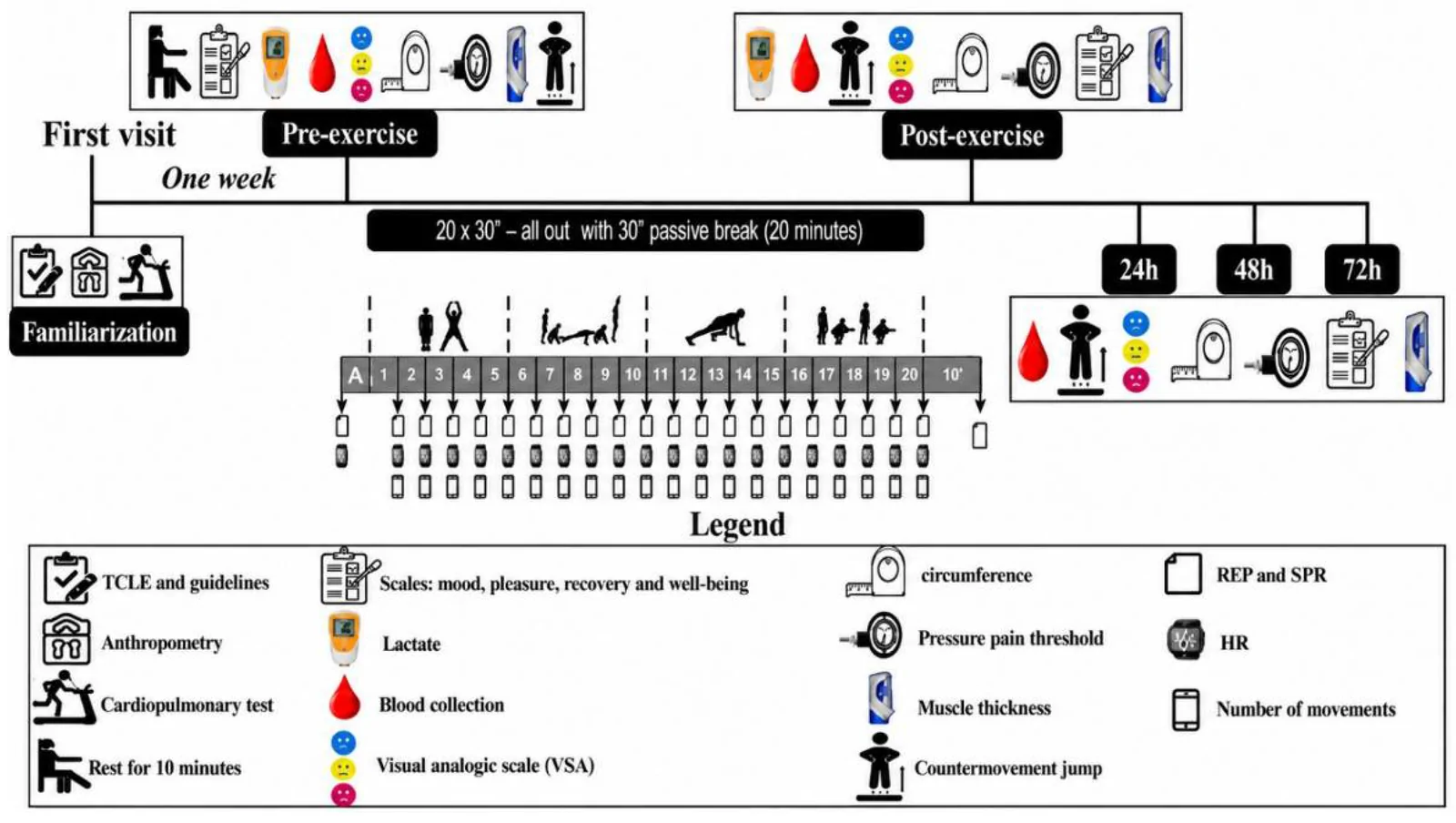
Representative scheme of the experimental design.

**Figure 2:**
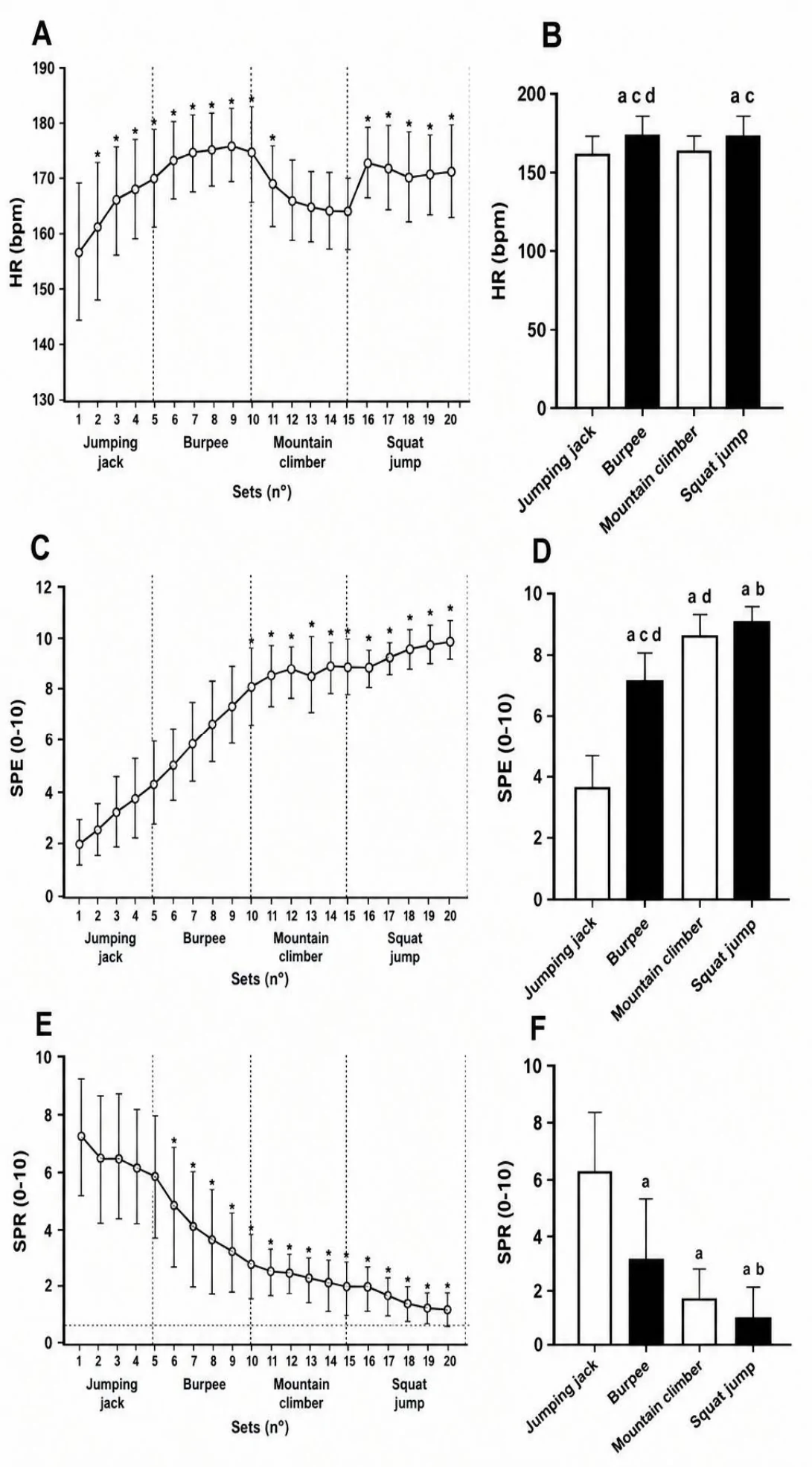
Values shown as the mean ± standard deviation of heart rate (Panel (**A**)), perceived effort (Panel (**C**)), perceived recovery (Panel (**E**)), and responses on exercise blocks, respectively (Panels (**B**,**D**,**F**)), based on the HIIT-C session. * Significant difference (*p* < 0.05) vs. 1st set. ^a^ Significant difference (*p* < 0.05) vs. jumping jacks. ^b^ Significant difference (*p* < 0.05) vs. burpees. ^c^ Significant difference (*p* < 0.05) vs. mountain climbing. ^d^ Significant difference (*p* < 0.05) vs. squat jumps.

**Figure 3:**
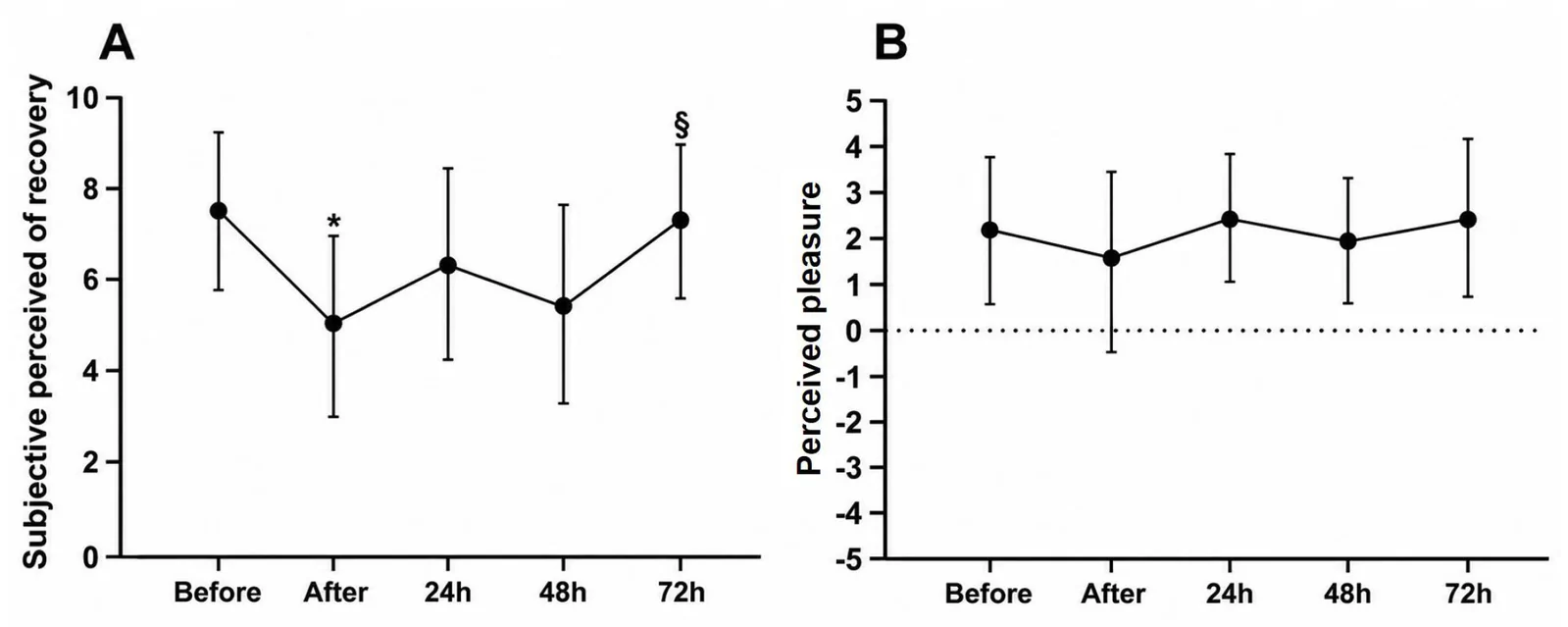
Values shown as the mean ± standard deviation of perceived recovery (Panel (**A**)) and pleasure (Panel (**B**)) before, after, and 24, 48, and 72 h after the HIIT-C session. * Significant difference (*p* < 0.05) vs. before. ^§^ Significant difference (*p* < 0.05) vs. after.

**Figure 4:**
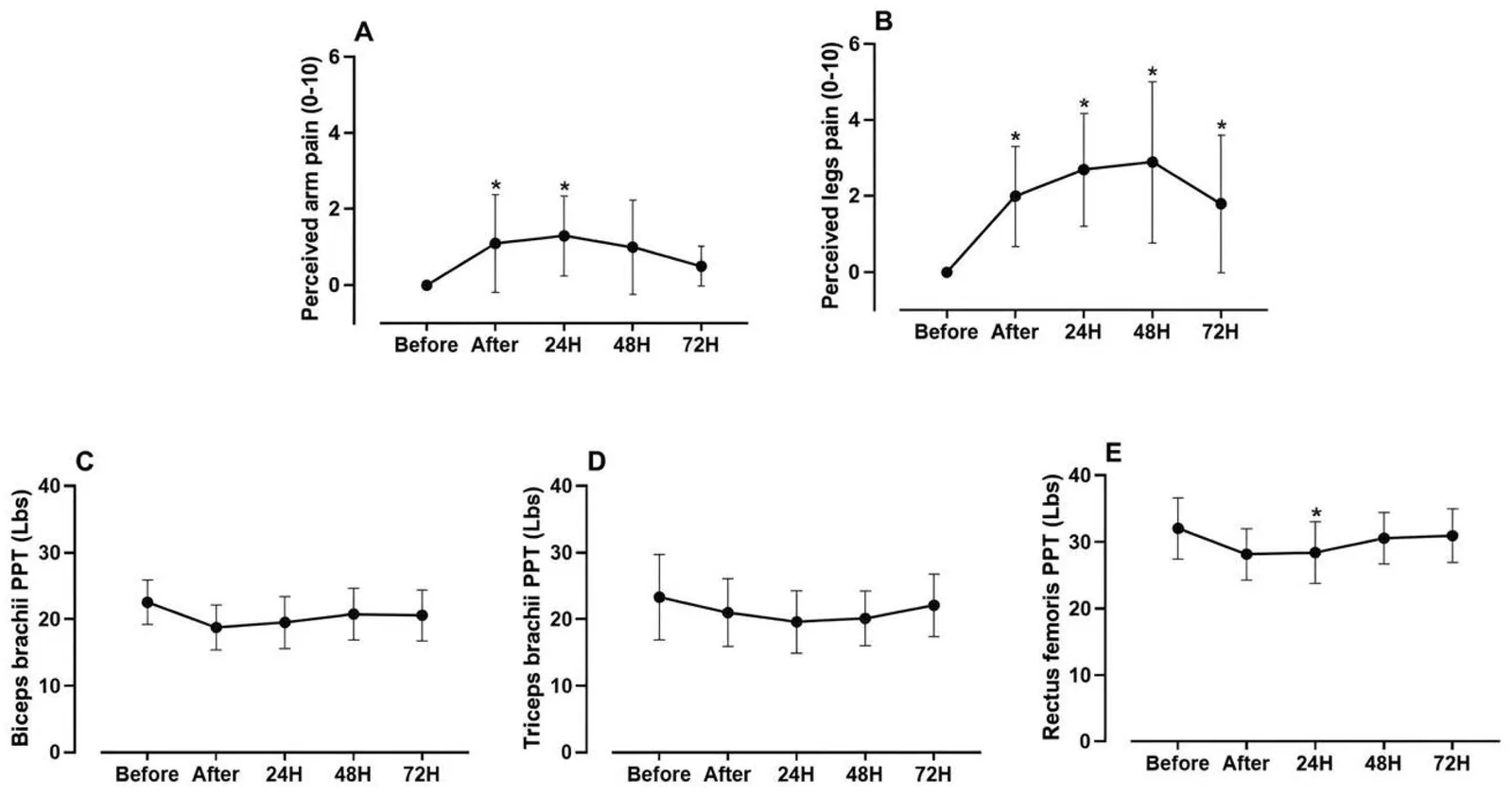
Values shown as the mean ± standard deviation of perceived pain in volunteers’ arms (Panel (**A**)) and legs (Panel (**B**)) and biceps brachii (Panel (**C**)), triceps brachii (Panel (**D**)), and rectus femoris (Panel (**E**)) pressure pain threshold (PPT) before, after, and 24, 48, and 72 h after the HIIT-C session. * Significant difference (*p* < 0.05) vs. before.

**Figure 5:**
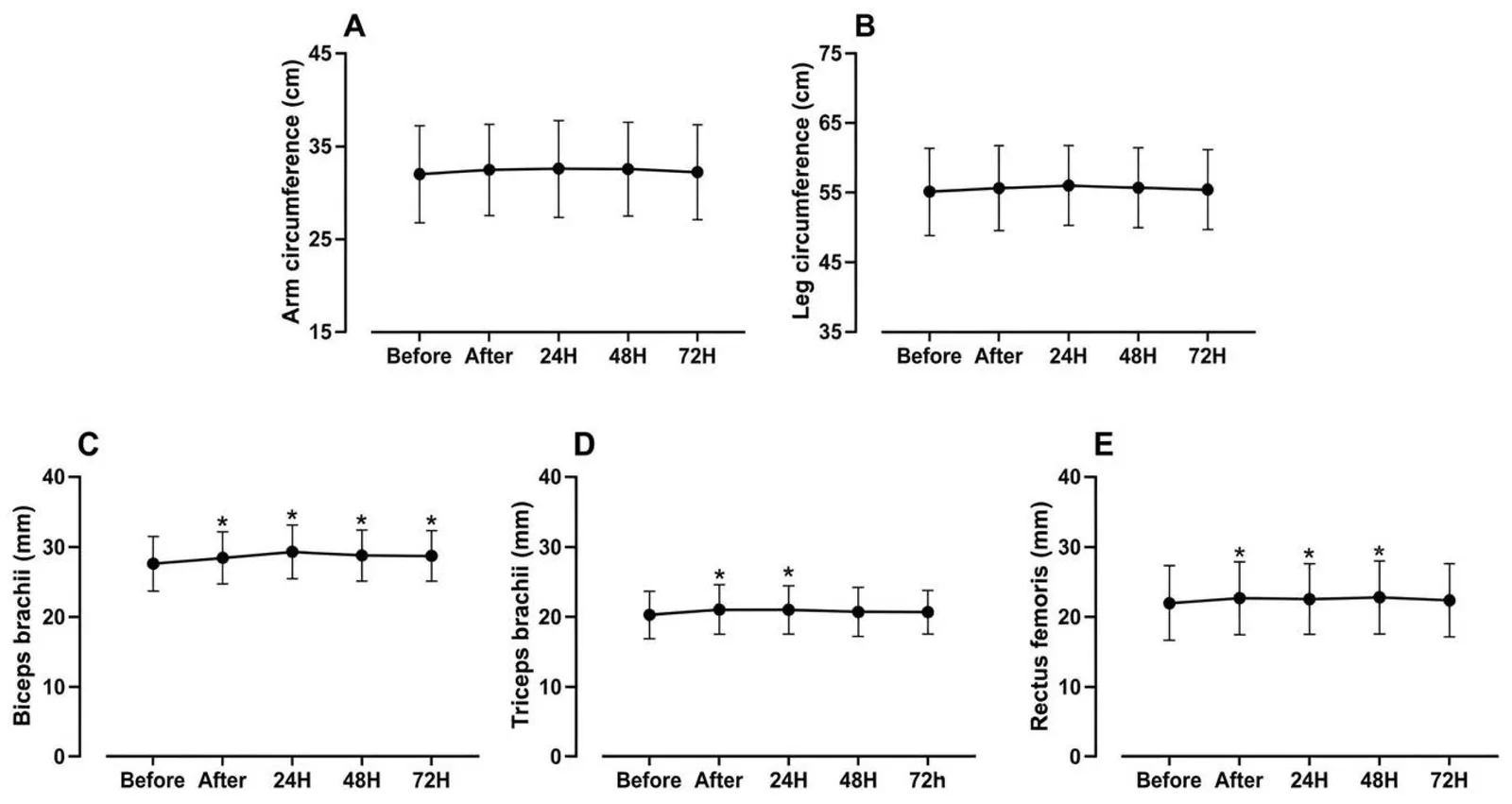
Values shown as the mean ± standard deviation of arm (Panel (**A**)) and leg (Panel (**B**)) circumferences and biceps brachii (Panel (**C**)), triceps brachii (Panel (**D**)), and rectos femoris muscular thickness (Panel (**E**)) before, after, and 24, 48, and 72 h after the HIIT-C session. * Significant difference (*p* < 0.05) vs. before.

**Figure 6:**
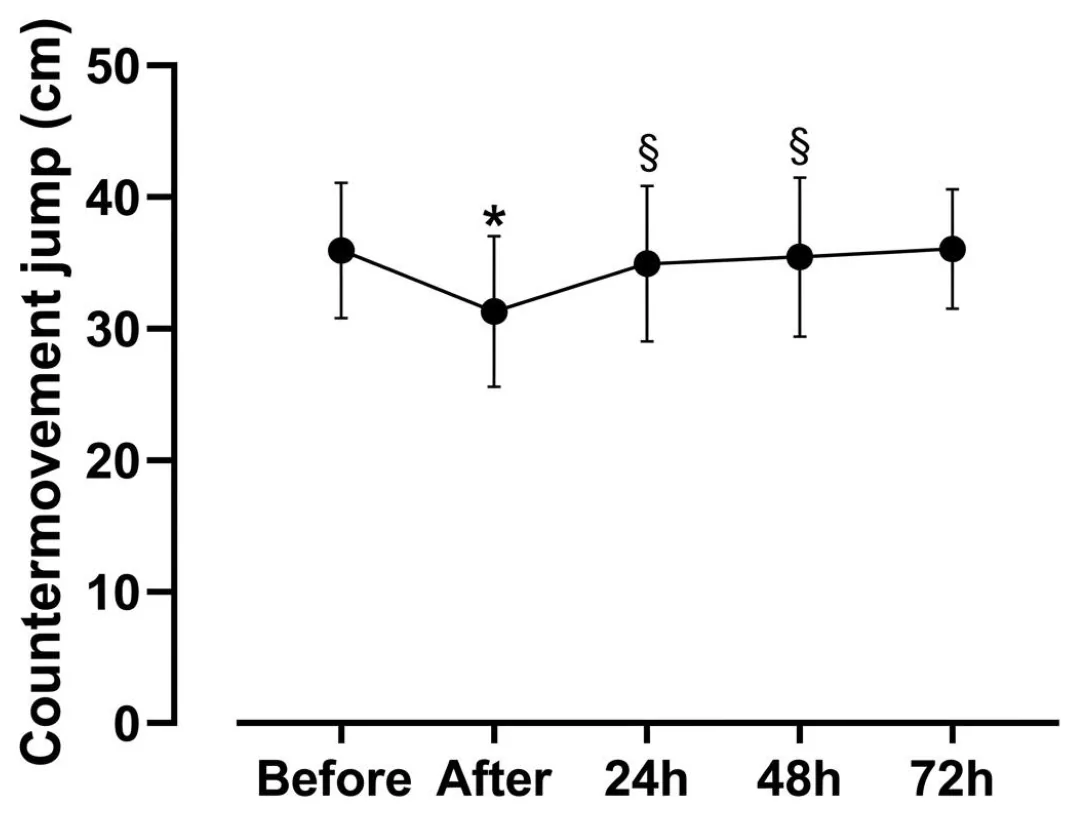
Values shown as the mean ± standard deviation of countermovement jumping before, after, and 24, 48, and 72 h after the HIIT-C session. * Significant difference (*p* < 0.05) vs. before. ^§^ Significant difference (*p* < 0.05) vs. after.

**Figure 7:**
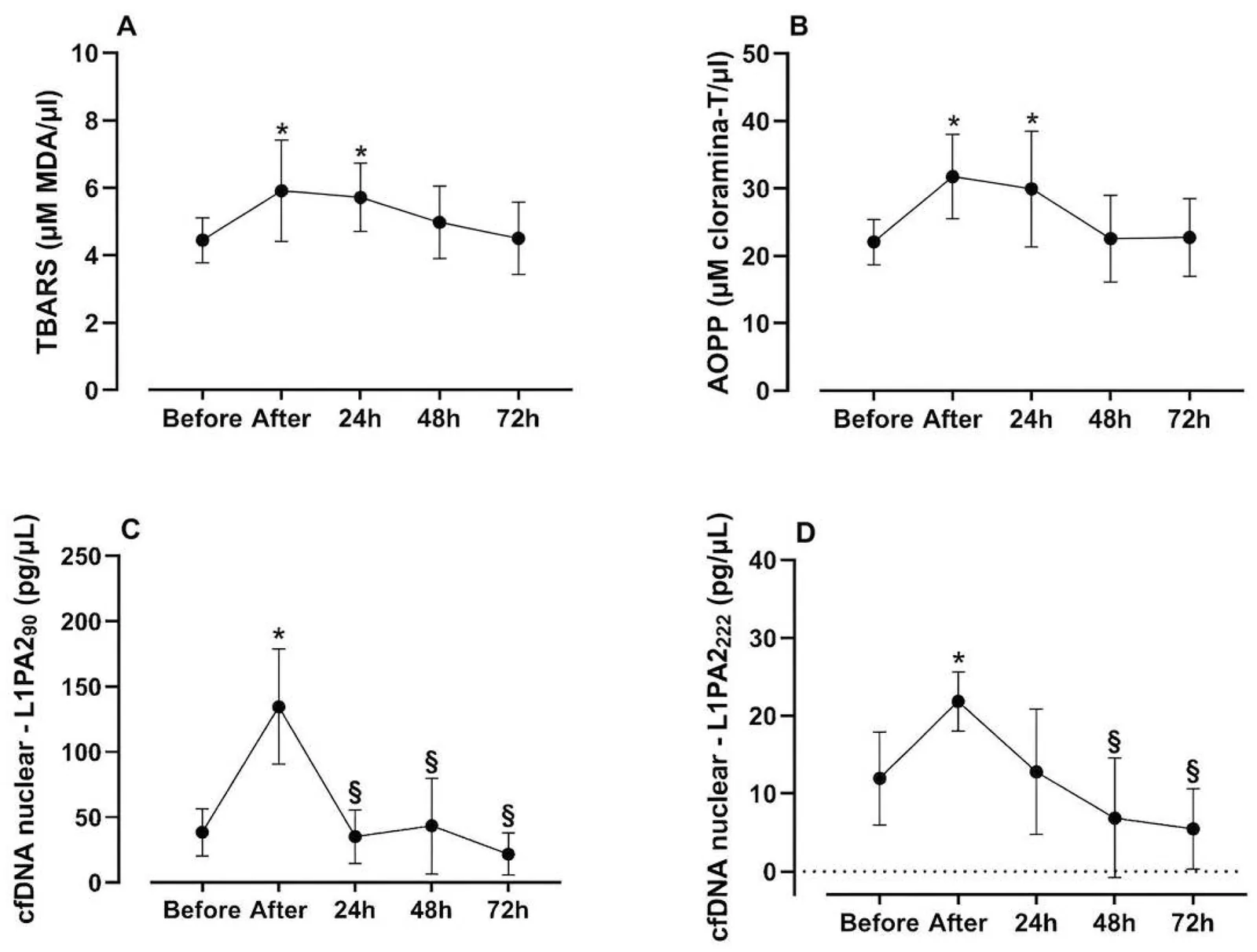
Values are shown as the mean ± standard deviation of thiobarbituric acid reactive substances–TBARS (Panel (**A**)), advanced protein oxidation products–AOPP (Panel (**B**)), cell-free DNA nuclear L1PA2_90_ (Panel (**C**)), and cell-free DNA nuclear L1PA2_222_ (Panel (**D**)) before, after and 24, 48, and 72 h after the HIIT-C session. * Significant difference (*p* < 0.05) vs. before. ^§^ Significant difference (*p* < 0.05) vs. after.

**Table 1:** General characteristics of the participants.

Characteristics	Mean ± SD	CV
Age (years)	25.2 ± 4.1	16.3%
Body mass (kg)	75.6 ± 12.2	16.1%
Height (m)	1.76 ± 0.03	1.54%
BMI (kg/m^2^)	24.4 ± 3.6	14.9%
∑SS (mm)	97.57 ± 38.8	42.4%
%F	12.56 ± 4.5	35.8%
FM (kg)	9.86 ± 4.7	47.5%
FFM (kg)	65.78 ± 8.6	13.1%
VO_2max_ (mL/kg/min)	47.6 ± 4.9	10.3%
HR_Max_	189.0 ± 8.9	4.7%
Physical activity time (min)	1174 ± 770.1	65.6%

Values are shown as means ± standard deviations. BMI: body mass index. ∑SS: sum of skinfolds. FM: fat mass, FFM: fat-free mass, VO_2max_: maximum oxygen volume uptake, HR_max_: maximum heart rate. CV: coefficient of variation.

**Table 2:** Training load parameters regarding the HIIT-C session.

Parameters	Before	After	MD	95% CI	ES	*p*
HR (bpm)	70.4 ± 4.5	169.7 ± 11.1	99.3 ± 12.4	90.5–108.1	11.7	<0.0001
Lactate (mmol/L)	1.2 ± 0.1	16.3 ± 2.3	15.1 ± 2.3	13.5–16.8	9.3	<0.0001
RPE (score 0–10)	0.9 ± 0.7	9.6 ± 0.3	8.7 ± 0.8	8.1–9.3	15.3	<0.0001
SPR (score 0–10)	10.0 ± 0.0	0.5 ± 0.5	9.4 ± 0.6	9.0–9.8	23.8	<0.0001

Values shown as the mean ± standard deviation of heart rate (HR), perceived exertion (RPE), and recovery (SPR). MD: mean difference. CI: confidence interval. ES: effect size.

**Table 3:** Response of mood and well-being indicators before and after the HIIT-C session.

Parameters	Before	After	24 h	48 h	72 h	*p*
**Mood**						
Tension	1.3 ± 1.8	0.7 ± 0.9	0.1 ± 0.3	0.3 ± 0.7	0 ± 0 *	=0.0077
Depression	0.5 ± 0.7	0.5 ± 0.7	0.2 ± 0.6	0.5 ± 0.7	0.2 ± 0.4	=0.4166
Anger	0 ± 0	0.3 ± 0.9	0 ± 0	0 ± 0	0 ± 0	=0.4060
Vigor	8.0 ± 3.4	4.7 ± 3.7	7.4 ± 2.5	5.6 ± 2.5 *	7 ± 2.9	=0.0317
Fatigue	1.2 ± 1.4	7.8 ± 3.8 *	2.3 ± 2.1 ^§^	2.5 ± 2.9 ^§^	1.4 ± 2.2 ^§^	<0.0001
Mental confusion	0.5 ± 0.7	1.6 ± 1.7	0 ± 0 ^§^	0 ± 0 ^§^	0.1 ± 0.3	=0.0087
Mood disturbance	95.5 ± 4.5	106.2 ± 8.4	95.2 ± 3.5 ^§^	97.7 ± 3.4 ^§^	94.7 ± 4.4 ^§^	=0.0013
**Well-being**						
Fatigue	4.0 ± 0.8	2.9 ± 1.2 *	3.2 ± 1.1	2.8 ± 1.0 *	3.4 ± 0.8	=0.0360
Sleep quality	4.1 ± 0.6	4.0 ± 0.8	4.0 ± 0.7	4.0 ± 0.9	4.3 ± 0.7	=0.5213
Muscle pain	4.2 ± 0.8	3.3 ± 0.9	3.0 ± 1.2	2.3 ± 1.0 *	3.2 ± 1.0	=0.0024
Stress level	4.0 ± 0.8	3.5 ± 0.8	3.6 ± 0.8	3.5 ± 0.7	3.5 ± 0.8	=0.4323
Mood	4.3 ± 0.5	4.0 ± 0.7	4.1 ± 0.9	3.9 ± 0.6	4.0 ± 0.7	=0.3315
General well-being	20.6 ± 2.8	17.7 ± 2.2	17.9 ± 3	16.5 ± 2.9 *	18.4 ± 2.7	=0.0071

Values shown as the mean ± standard deviation of mood and well-being before, after, and 24, 48, and 72 h after the HIIT-C session. * Significant difference (*p* < 0.05) vs. before moment. ^§^ Significant difference (*p* < 0.05) vs. after.

**Table 4:** Blood indicators of muscle damage before and after HIIT-C session.

Parameters	Before	After	24 h	48 h	72 h	*p*
CK (U/L)	271 ± 119	357 ± 145 *	442 ± 107 *	585 ± 107 *	483 ± 215	=0.0198
CK-MB (U/L)	34 ± 13	47 ± 16	38 ± 22	29 ± 19	42 ± 24	=0.1180
LDH (U/L)	296 ± 35	403 ± 69 *	362 ± 70	387 ± 92	395 ± 152	=0.0270
AST (U/L)	31 ± 9	69 ± 28 *	34 ± 9	28 ± 5	55 ± 33	=0.0022
ALT (U/L)	28 ± 14	33 ± 17 *	24 ± 9	21 ± 7	25 ± 7	=0.0318

Values shown as the mean ± standard deviation of creatine kinase (CK), cardiac creatine kinase (CK-MB), lactate dehydrogenase (LDH), aspartate aminotransferase (AST), and alanine aminotransferase (ALT) before, after, and 24, 48, and 72 h after the HIIT-C session. * Significant difference (*p* < 0.05) vs. before.

## Data Availability

The raw data supporting the conclusions of this article will be made available by the authors under reasonable request.
